# 

**Published:** 1879-02-20

**Authors:** 


RECEIPTED. 1878.—N. B. Richards; A. F. Pound; A. A. Lyon; W. T. Kendall; J. B. Payne; Geo. Redwood; J. B. Bailey; B. F. Phipps; C. H. Smith; J. E. Pope; R. D. Jackson; W. N. Ames; J. M. Pinkston; S. L. Dement.—1878 and 1879: R. H. Warren; W. K. Jones; J. W. Butts.—1879: E. A. Cox; L. K. Branch; W. S, Glass; J. A. McCallum; D. S. Williams; L. V. Weathers; J. N. Wadsworth, 6 months; E. W. Flemming; D. B. Hamilton; A. A. Hill; B. R. Doster; T. S. Parham; S. G. Hart; F. S. Wisdom; J. W. Hill; J. A. Williams, to June; J. M. Stansill; D. R. Fox; L. B. Bouchelle; E. H. Sherrell; H. T. Shiell, 6 months; T. B. Calling; Robert Kells; W. C. Jones; L. P. Coyner; Preston Pond; John Riches; John Gerdine; A. R. Wellborn, 6 months; J. A. Hayes; J. E. Martin; S. M. Hogan; B. F. Darnell; M. K. Harrison; W. J. Reddett; J. S. Sanders; A. A. Lyon; J. S. Bacon; L. Alison; J. R. Johnson; H. A. Hurst; C. F. Rodgers; T. D. Penn; A. G. Hobbs; T. L. Quillian; J. W. Thomas; J. C. Moody; Dr. McMikal; A. B. McWhorter; J. G. McCrary; W. B. King; J. T. Stephens; J. A. Holloway. J. F. Pou, '78; J. M. Boring; J. T. Stephens.

